# A survey of the availability, prices and affordability of essential medicines in Jiangsu Province, China

**DOI:** 10.1186/s12913-015-1008-8

**Published:** 2015-08-27

**Authors:** Xiaoyu Xi, Weixia Li, Jun Li, Xuan Zhu, Cong Fu, Xu Wei, Shuzhen Chu

**Affiliations:** School of International Pharmaceutical Business at China Pharmaceutical University, No.639 Longmian Street, Jiangning District, Nanjing, Jiangsu China

## Abstract

**Background:**

Field surveys conducted in China before the implementation of the essential medicine policy showed that Chinese individuals faced less access to essential medicines. This paper aims to evaluate the availability, prices and affordability of essential medicines in Jiangsu Province, China after the implementation of the policy in 2009.

**Methods:**

A cross-sectional survey was conducted in Jiangsu in 2013 using the World Health Organization/Health Action International (WHO/HAI) methodology. Data on the availability and prices of 50 essential medicines were collected from the public and private healthcare sectors.

**Results:**

The mean availabilities of innovator brands and lowest priced generics (LPGs) were 11.5 % and 100 % in primary healthcare facilities, 36.8 % and 32.6 % in the secondary and tertiary sectors, and 18.7 % and 42.9 % in the private sector, respectively. The median price ratios (MPRs) were 1.26 to 2.05 for generics and 3.76 to 27.22 for innovator brands. Treating ten common diseases with LPGs was generally affordable, whereas treatment with IBs was less affordable.

**Conclusions:**

The high availability of LPGs at primary healthcare facilities reflects the success of the essential medicine policy, while the low availability in secondary and tertiary levels and in private pharmacies reflects a failure to implement the policy in these levels. The health policy should be fully developed and enforced at the secondary and tertiary levels and in the private sector to ensure equitable access to health services.

**Electronic supplementary material:**

The online version of this article (doi:10.1186/s12913-015-1008-8) contains supplementary material, which is available to authorized users.

## Background

According to a report issued by the World Health Organization (WHO), approximately one-third of the global population lacks reliable access to needed medicines [1]. Access to healthcare is a fundamental human right that has been enshrined in international treaties and recognized by governments around the world. However, the fundamental right to health cannot be fulfilled without equitable access to essential medicines for priority diseases, particularly for people in developing countries with poor medical supplies [2].

China is also confronted with less access to essential medicines. A field survey conducted in Shandong and Gansu provinces in 2007 revealed that the median availability of surveyed medicines in hospital pharmacies ranged from 19 to 69 % [3]. Moreover, the Chinese people have suffered from inaccessible and unaffordable health services for decades. By the end of 2012, there were still 80,512,000 people, comprising approximately 6 % of the population, who lacked access to health services. In addition, government health expenditures accounted for approximately 5 % of GDP, with drug expenditures comprising up to 40 % of total health expenditures [4], among the highest proportions in the world [5]. To address the foregoing problems, the central government officially launched a new healthcare reform in 2009, one pillar of which was to establish a National Essential Medicine System (NEMS) to satisfy the public’s basic healthcare needs. The NEMS requires all primary health care institutions (namely, urban community health care centers, rural township hospitals, and village clinics) that receive government subsidies to stock and dispense all essential medicines at zero markup. Other types of medical organizations, such as secondary and tertiary hospitals and private hospitals, are expected to provide essential medicines as priority drugs for patients and ensure a certain use proportion that would vary according to local economic development [6]. Since the initiation of the NEMS in 2009, only one study has been conducted, in Shaanxi province in 2010, utilizing the Health Organization/Health Action International (WHO/HAI) methodology to review the prices, availability, and affordability of medicines [7]. Hence, this is the second study of this type since the essential medicine policy was implemented in China and the first conducted in Jiangsu province. The Essential Medicines List (EML) used in this survey is one latest Jiangsu Provincial EML in 2011, because for each province the EML is different.

Throughout China, public healthcare facilities are officially divided into three levels based on quality and levels of service: tertiary, secondary and primary. A higher level indicates better services. In addition, these facilities can be classified into public and private according to their nature. In China, patients can buy drugs from either public or private facilities. According to statistics from the provincial Department of Health in Jiangsu, annual per capita medical expenses paid by patients amounted to 221.2 CNY for outpatients and 10864.3 CNY for inpatients in 2013 and pharmaceutical expenditures accounted for 48.60 % and 43.24 %, respectively [8]. Therefore, it would be of strategic and practical significance to investigate whether the implementation of this health reform in Jiangsu has been effective. This study assesses medicine availability, prices and affordability in Jiangsu by collecting data from five of its cities. Particular attention is paid to the innovator brands (IBs) and lowest-priced generics (LPGs) available in Jiangsu and different types of medicine outlets (public hospitals and private pharmacies).

## Methods

We conducted a survey of the availability, prices and affordability of the essential medicines in Jiangsu, China by adopting the standardized WHO/HAI methodology [9, 10], which was modified as per the requirement of the study done at one province of China [11]. All data from this survey were collected from March 2013 to May 2013.

### Survey scope and medicine outlets

Jiangsu Province, located in eastern China, has a population of 78.98 million and 13 cities. Nanjing is the capital. Five representative cities of this province, rather than six as recommended by the WHO/HAI methodology, were selected as survey areas for data collection: Nanjing, Suzhou, Yangzhou, Suqian, and Yancheng. The selected cities are reachable within one day of travel from the capital and provide a large enough sample to represent the province.

In each survey area, the sample of public hospitals was identified by first selecting the main public hospital. An additional four public medicine outlets per survey area were then randomly selected from those within a four-hour drive from the main hospital. Most tertiary hospitals and medical resources are concentrated in the capital city, Nanjing, and few tertiary hospitals are in other cities. Therefore, 2 tertiary hospitals were selected in Nanjing, and no tertiary hospitals were selected in other cities. Additionally, there are relatively few primary healthcare facilities in Nanjing. Therefore, to make the sample more representative and consistent with the distribution of medical resources in Jiangsu Province, 2 tertiary and 3 secondary hospitals were selected in Nanjing, whereas 2 secondary and 3 primary hospitals were selected from each other cities. The public sector sample therefore contained five public medicine outlets in each of the five cities, yielding 25 public outlets. The sampled hospitals are all general, covering both urban and rural areas and serving approximately 142,290 patients on average each year. The private sector sample was determined by selecting the licensed private pharmacies closest to each of the selected public medicine outlets. Specifically, two private pharmacies near each secondary and tertiary healthcare facility were selected according to the recommendation of the methodology, but only one private pharmacy near each primary healthcare facility was selected due to the lack of nearby pharmacies, yielding 38 private outlets (See Table 1). The sample represented all retail pharmacies in each city. All the selected medicine outlets agreed to participate in the study, and a written informed consent form was signed before data collection.

**Table 1:** Main characteristics of the five selected survey areas in 2013

City/county	Nanjing (Capital)	Suzhou	Yangzhou	Suqian	Yancheng
Economic status	High-income	High-income	Medium-income	Low-income	Low-income
Geographic position	Southwest	Southeast	Center	Northwest	Northeast
NO. of Public hospitals	2 tertiary	2 secondary	2 secondary	2 secondary	2 secondary
	3 secondary	3 primary	3 primary	3 primary	3 primary
NO. of Private pharmacies	Two private pharmacies near the secondary and tertiary healthcare facilities and one private pharmacy near primary healthcare facilities				

### Selection of medicines

The WHO/HAI methodology requires a systematic survey of the prices of core and supplementary lists of medicines that are selected by each country according to local priorities in treating national health problems. Initially, 30 core medicines listed by WHO/HAI and 40 supplementary medicines were selected for inclusion in the survey. Following a pilot study, the list of medicines was modified because 7 core medicines were not on the EML, or not permitted to be used in general hospitals or sold in private pharmacies, and many supplementary medicines were also not on the list, or did not have reference prices. Hence, 50 medicines were finally selected for this survey, including 23 core medicines and 27 supplementary medicines. The former represent medicines commonly used in the treatment of a range of chronic and acute conditions, while the latter are of local importance [12]. The names and pack sizes of all medicines included on both lists (core and supplementary) are provided in Additional file 1. For each medicine, two forms (IBs and LPGs) were surveyed. The manufacturers of IBs were easily identified because each IB has a unique manufacturer, while those of LPGs were determined during data collection at each facility.

### Data collection

Prior to data collection, a one-week training was held to provide area supervisors, data collectors and data entry personnel with the knowledge and skills required to conduct the medicine availability, prices and affordability survey in an accurate and reliable manner. A standardized data collection form was used to ensure data reliability and consistency. Additionally, a localized pilot study was conducted during the training in Nanjing to verify the feasibility and effectiveness of the survey.

Area supervisors made appointments with responsible persons at the target healthcare centers before formal data collection began to make it convenient for researchers to collect information. Then, data collectors visited each region separately to finish collection, and area supervisors met with them in the field at the end of each day to check completed data collection forms and ensure that the data were complete, consistent and legible. Data on the availability and patient prices of medicines at the selected public hospitals and private pharmacies were collected. The data collection was completed within six weeks. Because human and animal clinical trials were not involved in this study, the need for ethical review was waived by the Institutional Review Board of China Pharmaceutical University, China. This research adhered to the STROBE guidelines for observational studies (see Additional file 2).

### Data entry and analysis

Data entry and analysis was facilitated by a standardized computerized workbook for double entry to record the data generated by the survey. We verified the data before the unit prices were entered into the computerized workbook provided by WHO/HAI [13]. All data were entered twice, followed by software verification and validation through “double entry” and “data checker” functions to identify data entry errors. Furthermore, codes, instead of the actual names, were used to identify these public healthcare facilities and retail pharmacies to maintain their anonymity.

Information on the availability and prices of medicines in different geographic areas and healthcare sectors was generated using the same software employed for data analysis. The study endpoints focused on three measures: medicine availability, prices and affordability. Availability was defined as the proportion of pharmacies in which the medicines were available at the time of the survey. The availabilities of both types of medicines (IBs and LPGs) in public hospitals and private pharmacies were calculated. Prices were presented as median price ratios (MPR) in this study. The MPR is the ratio of the median local unit price across facilities divided by the median international reference unit price (IRP). In this survey, medicine prices from the *Drug Prices Guide in 2011* issued by Management Science for Health (MSH) were adopted as the IRPs for core medicines, but because MSH prices were not available for most supplementary medicines, Spanish manufacturers’ selling prices were used as their reference prices (supplied by WHO/HAI project member Carmen Peres-Casas). Spain is known to have relatively low manufacturer selling prices. MPRs were only calculated if the medicine was available at a minimum of four facilities. For the purposes of this discussion, we use the following MPR cut-off points for patient prices to represent acceptable local price ratios: MPR ≤ 1.5 for public hospital patient prices and MPR ≤ 2.5 for retail pharmacies patient prices [14]. Affordability was estimated by comparing the total cost of a medicine for a standard course of treatment to the daily wage of the lowest paid unskilled government worker, which was 42.7 CNY per day at the time of the survey [15].

## Results

### Availability

Public hospitals in this survey are divided into two categories: primary healthcare facilities and secondary and tertiary healthcare facilities. Table 2 shows the mean availability for public hospitals and retail pharmacies. The availability of LPGs was 100 % at the primary facilities, which was attributed to the NEMS requirements to be stock all essential medicines. For IBs, however, only 11.5 % were available in primary facilities, and only four medicines—Amlodipine, Captopril, Ciprofloxacin and Metformin—had >50 % availability.

**Table 2:** Availability of medicines in public hospitals and retail pharmacies

	Primary healthcare facilities (*n* = 12)	Secondary and tertiary healthcare facilities (*n* = 13)	Retail pharmacies (*n* = 38)
IB	11.50 %	36.80 %	18.70 %
LPG	100.00 %	32.60 %	42.90 %

*IB*, Innovator Brand

*LPG*, Lowest Priced Generic

The data also showed that the mean availability of LPGs and IBs was 32.6 % and 36.8 %, respectively, in secondary and tertiary facilities. Additionally, the mean availability of LPGs and IBs was 42.9 % and 18.7 %, respectively, in retail pharmacies (see Table 2). The availability of LPGs in these two types of outlets was lower than that in primary facilities (100 %). In fact, only 7 LPGs in the secondary and tertiary facilities and 20 in the retail pharmacies had >50 % availability. Additionally, only 24 IBs in the secondary and tertiary facilities and 4 in the retail pharmacies had >50 % availability. Although the mean availability of IBs in the secondary and tertiary facilities (36.8 %) was low, this value was the highest of the surveyed sectors. Overall, the highest availability of LPGs (100 %) and the lowest of IBs (11.5 %) were observed at primary healthcare facilities, whereas the IBs were most available (36.8 %) and LPGs least available (32.6 %) at secondary and tertiary facilities.

### Patient prices

As shown in Table 3, the MPRs of all LPGs ranged from 1.26 to 2.05, which demonstrated that the patient prices of LPGs approached the acceptable price line and were similar to the IRPs. However, the patient prices of IBs were all above the threshold level, with MPRs ranging from 3.76 to 27.22. Moreover, the MPRs of most IBs in public sector outlets were much higher (approximately 2 to 7 times higher) than those in private pharmacies were.

**Table 3:** MPRs of survey medicines in public hospitals and retail pharmacies

	MPRs of core medicines		MPRs of supplementary medicines	
	IB	LPG	IB	LPG
Primary healthcare facilities (*n* = 12)	3.76	1.26	27.22	1.66
Secondary and tertiary healthcare facilities (*n* = 13 )	6.78	1.98	16.72	2.05
Retail pharmacies (*n* = 38)	4.13	1.81	4.01	1.82

One outlier drug was nifedipine sustained-release. This drug, either in the form of LPGs or IBs, had high median prices of, on average, 4 to 17 times the IRP in all sectors. The greatest price difference (27.22 times the IRP) was found for the IBs of supplementary medicines in primary healthcare facilities.

### Affordability

In view of the low availability of core medicines, we considered ten priority diseases to measure the affordability of standard treatments. As shown in Table 4, the cost of purchasing LPGs at all surveyed was between 0.1 and 0.8 days’ wages, which indicated that generic medicines in Jiangsu Province were fairly affordable. The most affordable LPGs were glibenclamide for treating diabetes and atenolol for treating hypertension (which cost 0.1 days’ wages in all sectors). IBs, however, were less affordable. When IBs were prescribed and dispensed in the surveyed sectors, some treatments were surprisingly costly. For example, treating hypercholesterolemia with IB simvastatin would cost over 7 days’ wages, while treating arthritis with IB diclofenac would cost 2 days’ wages. Overall, IB products were less affordable than LPGs in both the public and private sectors.

**Table 4:** Affordability of core medicines for common diseases in public hospitals and retail pharmacies

Treatment	Type	The primary healthcare facilities		The secondary and tertiary facilities		Retail pharmacies	
		Median price	Day’s wages	Median price	Day’s wages	Median price	Day’s wages
		(Yuan)		(Yuan)		(Yuan)	
Asthma:	IB	NA	NA	35.31	1.10	NA	NA
Salbutamol inhaler	LPG	15.33	0.50	21.80	0.70	18.55	0.60
0.1 mg/dose*200 doses							
Diabetes:	IB	NA	NA	6.87	0.20	NA	NA
GlIBenclamide	LPG	1.75	0.10	3.05	0.10	2.61	0.10
5 mg*2*30 days							
Hypertension:	IB	NA	NA	NA	NA	NA	NA
Atenolol	LPG	3.78	0.10	4.62	0.10	4.17	0.10
50 mg*1*30 days							
Hypertension:	IB	13.66	0.40	9.71	0.30	17.66	0.60
Captopril	LPG	7.84	0.30	9.71	0.30	8.74	0.30
25 mg*2*30 days							
Hypercholesterola emia:	IB	228.71	7.40	336.19	10.80	221.58	7.10
Simvastatin	LPG	12.80	0.40	NA	NA	25.65	0.80
20 mg*1*30 days							
Depression:	IB	NA	NA	20.02	0.70	18.98	0.70
Amitriptyline	LPG	11.90	0.40	NA	NA	13.65	0.50
25 mg*3*30 days							
Adult respiratory infection:	IB	11.90	0.40	14.36	0.50	12.63	0.40
Ciprofloxacin	LPG	5.03	0.20	NA	NA	5.54	0.20
500 mg*2*7 days							
Adult respiratory infection:	IB	NA	NA	45.39	1.50	43.28	1.40
Amoxicillin	LPG	20.94	0.70	23.68	0.80	22.39	0.80
500 mg*3*7 days							
Arthritis:	IB	NA	NA	62.40	2.00	NA	NA
Diclofenac	LPG	15.89	0.60	15.89	0.60	14.36	0.50
50 mg*2*30 days							
Ulcer:	IB	24.28	0.80	28.34	0.90	25.93	0.80
Omeprazole	LPG	10.43	0.30	12.65	0.40	11.55	0.40
20 mg*1*30 days							

Note: NA, not available

## Discussion

Our results indicate, first, the success of the essential medicine policy because the availability of LPGs in primary care facilities has reached 100 %. According to statistics from the Ministry of Health in China, the visits to primary outlets has reached 3.92 billion by November 2014, accounting for 57.9 % of the total visits. Therefore, the success has enabled the population who visit primary care facilities for common ailments to have reliable access to essential medicines after 2009. However, the availability of LPGs in secondary and tertiary facilities and private sectors remains relatively low (32.6 % and 42.9 % respectively). Minimal changes can be observed when compared the availabilities of LPGs in secondary and tertiary facilities for 21 common medicines surveyed in Shandong before the reform (28.82 %) [16] and in this survey after the reform (30.53 %), which reflects a failure to implement the policy in these levels. Three reasons might explain for the finding. First, secondary and tertiary facilities and private sector outlets are not willing to procure cheap LPGs. The “drug-maintaining-medicine” system in China permits hospitals to add a 15 % markup to the wholesale prices of drugs. Therefore, the lower the procurement price, the lower the revenue from the markup. While private pharmacies are difficult to maintain operations by selling overmuch LPGs. Second, pharmaceutical enterprises might be reluctant to promote LPGs to secondary and tertiary facilities and private sectors as well. LPGs are regulated by the NEMS to maintain low prices, which imply low profit margins, yet massive marketing expenses are necessitated to propel the use of them in these sectors. Third, physician inclination to prescribe IBs is also a critical factor. Physicians believe that the quality and efficacy of IBs are higher than those of LPGs are. Admittedly, physicians can also obtain sizeable commissions from medical representatives on prescriptions of IBs. According to a study, 80 % of medicines in China are sold in hospitals [17], suggesting that public hospitals are core players in the medicine supply chain. Hence, the availability of LPGs in secondary and tertiary facilities must be improved. Essential medicines are expected to be preferentially stock and used in public hospitals.

Low availability of IBs can be observed in both public and private sectors as well, particularly in primary healthcare facilities and private pharmacies. Limited access to IBs in primary facilities can be attributed to Chinese health policy to increase the proportion of LPGs in primary facilities, to decrease health expenditures and to improve drug affordability for the public, whereas the low availability of IBs in private pharmacies conforms to patients’ consumption habits. Because purchasing most IBs from private pharmacies requires a physician’s prescription in China, patients prefer to obtain drugs directly from public hospitals when seeing a physician.

To address the poor availability of both IBs and LPGs in secondary care, tertiary care and private facilities, a more specific guidance for preferential use of essential medicines in these facilities should be issued by the government to improve people’s access to them. Furthermore, mechanisms for reimbursement of the use of essential medicines in secondary, tertiary and private levels from the government are indispensable to ensure a much higher availability as well. Most importantly, the “drug-maintaining-medicine” system prevalent in these years should be eliminated in secondary and tertiary sectors to enhance physicians’ willingness to procure and prescribe LPGs, while additional medicine service fees and more government investment can be alternatives to keep these hospitals running.

Little difference is found among medicine outlets when comparing the prices of LPGs to their IRPs. Because LPGs of the same medication do not vary obviously in quality or efficacy and are manufactured by numerous pharmaceutical firms in China, these enterprises depend on price advantages to survive under the tremendous pressure of market competition. Thus, the prices of LPGs are low. Only one medication, nifedipine sustained-release, was found to have a much higher price than its IRP in all surveyed sectors. The higher price of this drug is due to its sustained-release function, which ordinary nifedipine does not possess, and the lack of competition in the market.

The MPRs of medications on the core list of IBs available in both sectors were generally higher in the public sector than in the private sector (median MPRs were 6.78 and 4.13 for the public and private sectors, respectively). This finding is similar to that in the studies conducted in Hubei and Shaanxi (core list MPRs were 1.05 and 1.84 for the public sector and 0.51 and 1.46 for the private sector in Hubei and Shaanxi, respectively) [7, 18]. The high prices of IBs in the public sector can be attributed to the abovementioned “drug-maintaining-medicine” system. Though the Chinese government has released a varied mix of policies designed to reduce drug prices over the past decade, no substantial effects have been produced [19]. On the contrary, private pharmacies choose to reduce medicine prices to attract customers and remain competitive in the pharmaceutical market; hence, the price differences increase. In Thailand, analyses revealed that the MPR for IBs was higher in the private sector (11.60) than in the public sector (4.36) [20]. This difference in prices might be a reason for the difference in the healthcare policies of Thailand and China.

Regarding the interpretation of affordability, caution should be exercised when extrapolating the findings to the national level because there may be regional differences in affordability due to differences economic development across the country. The data from this survey show that most LPGs for standard treatments are affordable; this affordability can be explained by the increased living standards of residents, which enable them to afford high medical expenses. Statistics show that only 2 % of the population in Jiangsu lives below the poverty line, a proportion ranking third in China and higher than the national average. Moreover, full medical insurance coverage in Jiangsu Province also protects residents from the high costs of obtaining medical services. A comparative analysis of the affordability of IBs and LPGs indicates that the former are less affordable than the latter. Though IBs will gradually lose their market competitiveness given the increasing prevalence of essential medicines, their brand status is not replaceable over a relatively short period. Thus, it is indispensable to continue to pay attention to the affordability of IBs.

The present study has two limitations. First, the data are available only for the day they were collected at each facility in five cities of Jiangsu Province and may not reflect average monthly or yearly availability of medicines. Another is that calculating affordability based on unskilled government worker wages may lead to overly optimistic results because a portion of the national population earns less than that wage.

## Conclusions

In Jiangsu Province, the high availability of LPGs in primary care facilities reflects the success of the government’s drug policy, whereas their low availability in secondary and tertiary levels and private pharmacies reflects failure to implement this policy at these levels. The national essential medicine policy should be fully developed and enforced at the secondary and tertiary levels and in the private pharmacies of the Chinese health system to ensure equitable access to basic health services, particularly for the poor.

## Additional files

Additional file 1:
**List of medicines surveyed in five regions in 2013.** List of the medicines investigated (*N* = 50) from both the core and supplementary lists, including their names, strengths, dosage forms and pack sizes recommended. (PDF 176 kb) Additional file 2:
**Completed checklist for the STROBE guidelines for observational studies.** A completed checklist for the STROBE guidelines for observational studies, showing that this research adhered to the guidelines. (PDF 193 kb)

## Abbreviations

NEMS: National Essential Medicine System
EML: Essential Medicines List
IBs: Innovator brands
LPGs: Lowest-priced generic products
MPR: Median price ratio
IRP: International reference price
MSH: Management Science for Health
