# Supplementary material for: A survey of the availability, prices and affordability of essential medicines in Jiangsu Province, China
Source: BMC Health Serv Res. 2015 Aug 27;15:345. doi: 10.1186/s12913-015-1008-8 (PMC4549946; doi:10.1186/s12913-015-1008-8)
Supplement: Additional file 1: — List of medicines surveyed in five regions in 2013. List of the medicines investigated (N = 50) from both the core and supplementary lists, including their names, strengths, dosage forms and pack sizes recommended. (PDF 176 kb) [file 12913_2015_1008_MOESM1_ESM.pdf]

Additional file 1 List of medicines surveyed in five regions in 2013

Med. No.	Medicine Name	Medicine Strength	Dosage Form	Pack Size Recommended	Medicine list	EML
1	Acetazolamide	250mg	cap/tab	100	Supplementary	yes
2	Albendazole	200mg	cap/tab	2	Core	yes
3	Amitriptyline	25 mg	cap/tab	100	Core	yes
4	Amlodipine	5mg	cap/tab	30	Core	yes
5	Amoxicillin	500 mg	cap/tab	21	Core	yes
6	Atenolol	50 mg	cap/tab	60	Core	yes
7	Azathioprine	50mg	cap/tab	100	Supplementary	yes
8	Azithromycin	250mg	cap/tab	6	Supplementary	yes
9	Bisacodyl	5mg	cap/tab	8	Supplementary	yes
10	Captopril	25 mg	cap/tab	60	Core	yes
11	Carbamazepine	100mg	cap/tab	100	Supplementary	yes
12	Ceftazidime	0.03mg	disc		Supplementary	yes
13	Cefuroxime	750mg	vial	1	Supplementary	yes
14	Cephalexin	250mg	cap/tab	28	Core	yes
15	Ciprofloxacin	500 mg	cap/tab	10	Core	yes
16	Clarithromycin	250mg	cap/tab	12	Supplementary	yes

17	Dexamethasone	0.1%opht	drop	5	Supplementary	yes
18	Diazepam	5 mg	cap/tab	100	Core	yes
19	Diclofenac	50 mg	cap/tab	100	Core	yes
20	Digoxin	250mg	cap/tab	100	Supplementary	yes
21	Enalapril	10mg	cap/tab	30	Core	yes
22	Erythromycin	250mg	cap/tab	20	Supplementary	yes
23	Fluconazole	150mg	cap/tab	7	Supplementary	yes
24	Fluoxetine	20mg	cap/tab	30	Core	yes
25	Folic Acid	1mg	cap/tab	31	Supplementary	yes
26	Furosemide	40mg	cap/tab	20	Supplementary	yes
27	Glibenclamide	5 mg	cap/tab	60	Core	yes
28	Gliclazide	80mg	cap/tab	100	Core	yes
29	Hydrochlorothiazide	25mg	cap/tab	30	Core	yes
30	Hydrocortisone	1%	cream	10	Supplementary	yes
31	Ibuprofen	400mg	cap/tab	30	Core	yes
32	Indometacin	25mg	cap/tab	100	Supplementary	yes
33	Insulin	100iu/ml	vial	4	Supplementary	yes
34	Ketoconazole	200mg	cap/tab	10	Supplementary	yes
35	Levofloxacin	250mg	cap/tab	21	Supplementary	yes

36	Metformin	500mg	cap/tab	100	Core	yes
37	Metoclopramide	10mg	cap/tab	100	Supplementary	yes
38	Metronidazole	200mg	cap/tab	28	Core	yes
39	Nifedipine sustained - release	20mg	cap	30	Core	yes
40	Norfloxacin	400mg	cap/tab	20	Supplementary	yes
41	Ofloxacin	200mg	cap/tab	30	Supplementary	yes
42	Omeprazole	20 mg	cap/tab	30	Core	yes
43	Pethidine	50mg/ml	ampoule	5	Supplementary	yes
44	Phenytoin	100mg	cap/tab	200	Supplementary	yes
45	Promethazine	25mg	cap/tab	20	Supplementary	yes
46	Ranitidine	150mg	cap/tab	60	Core	yes
47	Rifampicin	150mg	cap/tab	100	Supplementary	yes
48	Salbutamol inhaler	100 mcg/dose	dose	200	Core	yes
49	Simvastatin	20 mg	cap/tab	30	Core	yes
50	Tetanus Antitoxin	1500 iu	ampoule		Supplementary	yes

*EML，Essential Medicines List

List of the medicines investigated (N=50) from both the core and supplementary lists, including

their names, strengths, dosage forms and pack sizes recommended.
