# Supplementary material for: A survey of the availability, prices and affordability of essential medicines in Jiangsu Province, China
Source: BMC Health Serv Res. 2015 Aug 27;15:345. doi: 10.1186/s12913-015-1008-8 (PMC4549946; doi:10.1186/s12913-015-1008-8)
Supplement: Additional file 2: — Completed checklist for the STROBE guidelines for observational studies. A completed checklist for the STROBE guidelines for observational studies, showing that this research adhered to the guidelines. (PDF 193 kb) [file 12913_2015_1008_MOESM2_ESM.pdf]

Additional file 2 Completed checklist for the STROBE guidelines for observational studies

	Item No.	Recommendation	Page No.	Relevant text from manuscript
**Title and abstract**	1	(*a*) Indicate the study's design with a commonly used term in the title or the abstract	1	A survey of the availability, prices and affordability of essential medicines in Jiangsu Province, China
		(*b*) Provide in the abstract an informative and balanced summary of what was done and what was found	2	A cross-sectional survey was conducted in Jiangsu in 2013 using the World Health Organization/Health Action International (WHO/HAI) methodology.<br>The high availability of LPGs at primary healthcare facilities reflects the success of the essential medicine policy, while the low availability in secondary and tertiary levels and in private pharmacies reflects a failure to implement the policy in these levels.
**Introduction**				
Background/rationale	2	Explain the scientific background and rationale for the investigation being reported	3,4	China is confronted with less access to essential medicines.<br>Moreover, the Chinese people have suffered from inaccessible and unaffordable health services for decades.<br>In addition, government health expenditures accounted for approximately 5% of GDP, with drug expenditures comprising up to 40% of total health expenditures, among the highest proportions in the world.
Objectives	3	State specific objectives, including any prespecified hypotheses	5	This study assesses medicine availability, prices and affordability in Jiangsu by collecting data from five of its cities. Particular attention is paid to the innovator brands (IBs) and lowest-priced generics (LPGs) available in Jiangsu and different types of medicine outlets (public hospitals and private pharmacies).
**Methods**				
Study design	4	Present key elements of study design early in the paper	5	We conducted a survey of the availability, prices and affordability of the essential medicines in Jiangsu, China by adopting the standardized WHO/HAI methodology, which was modified as per the requirement of the study done at one province of China.
Setting	5	Describe the setting, locations, and relevant dates, including periods of recruitment, exposure, follow-up, and data collection	5,6,8	All data from this survey were collected from March 2013 to May 2013. Jiangsu Province, located in eastern China, has a population of 78.98 million and 13 cities. Nanjing is the capital. Five representative cities of this province, rather than six as recommended by the WHO/HAI methodology, were selected as survey areas for data collection: Nanjing, Suzhou, Yangzhou, Suqian, and Yancheng.

				Additionally, a localized pilot study was conducted during the training in Nanjing to verify the feasibility and effectiveness of the survey.<br>Data on the availability and patient prices of medicines at the selected public hospitals and private pharmacies were collected.
Participants	6	Give the eligibility criteria, and the sources and methods of selection of participants	5,6,7	Prior to data collection, a one-week training was held to provide area supervisors, data collectors and data entry personnel with the knowledge and skills required to conduct the medicine availability, prices and affordability survey in an accurate and reliable manner.
Variables	7	Clearly define all outcomes, exposures, predictors, potential confounders, and effect modifiers. Give diagnostic criteria, if applicable	9	The study endpoints focused on three measures: medicine availability, prices and affordability. Availability was defined as the proportion of pharmacies in which the medicines were available at the time of the survey. MPR is the ratio of the median local unit price across facilities divided by the median international reference unit price (IRP).<br>Affordability was estimated by comparing the total cost of a medicine for a standard course of treatment to the daily wage of the lowest paid unskilled government worker.
Data sources/ measurement	8*	For each variable of interest, give sources of data and details of methods of assessment (measurement). Describe comparability of assessment methods if there is more than one group	8,9,10	Data on the availability and patient prices of medicines at the selected public hospitals and private pharmacies were collected.<br>In this survey, medicine prices from the *Drug Prices Guide in 2011* issued by Management Science for Health (MSH) were adopted as the IRPs for core medicines, but because MSH prices were not available for most supplementary medicines, Spanish manufacturers' selling prices were used as their reference prices (supplied by WHO/HAI project member Carmen Peres-Casas).<br>Affordability was estimated by comparing the total cost of a medicine for a standard course of treatment to the daily wage of the lowest paid unskilled government worker, which was 42.7 CNY per day at the time of the survey.
Bias	9	Describe any efforts to address potential sources of bias	9	All data were entered twice, followed by software verification and validation through "double entry" and "data checker" functions to identify data entry errors. Furthermore, codes, instead of the actual names, were used to identify these public healthcare facilities and retail pharmacies to maintain their anonymity.
Study size	10	Explain how the study size was arrived at	5,6,7	Five representative cities of this province, rather than six as recommended by the WHO/HAI methodology, were selected as survey areas for data collection: Nanjing, Suzhou, Yangzhou, Suqian, and Yancheng. The selected cities are reachable within one day of travel from the capital and provide a large enough sample to represent the

				province.<br>The public sector sample therefore contained five public medicine outlets in each of the five cities, yielding 25 public outlets.<br>The private sector sample was determined by selecting the licensed private pharmacies closest to each of the selected public medicine outlets.<br>Hence, 50 medicines were finally selected for this survey, including 23 core medicines and 27 supplementary medicines.
Quantitative variables	11	Explain how quantitative variables were handled in the analyses. If applicable, describe which groupings were chosen and why	9	The availabilities of both types of medicines (IBs and LPGs) in public hospitals and private pharmacies were calculated.<br>Prices were presented as median price ratios (MPR) in this study. The MPR is the ratio of the median local unit price across facilities divided by the median international reference unit price (IRP). In this survey, medicine prices from the *Drug Prices Guide in 2011* issued by Management Science for Health (MSH) were adopted as the IRPs for core medicines, but because MSH prices were not available for most supplementary medicines, Spanish manufacturers' selling prices were used as their reference prices (supplied by WHO/HAI project member Carmen Peres-Casas).<br>Affordability was estimated by comparing the total cost of a medicine for a standard course of treatment to the daily wage of the lowest paid unskilled government worker, which was 42.7 CNY per day at the time of the survey.
Statistical methods	12	(*a*) Describe all statistical methods, including those used to control for confounding	9	All data were entered twice, followed by software verification and validation through "double entry" and "data checker" functions to identify data entry errors.
		(*b*) Describe any methods used to examine subgroups and interactions	NA	NA
		(*c*) Explain how missing data were addressed	NA	NA
		(*d*) *Cross-sectional study*—If applicable, describe analytical methods taking account of sampling strategy	5,6	The selected cities are reachable within one day of travel from the capital and provide a large enough sample to represent the province.<br>An additional four public medicine outlets per survey area were then randomly selected from those within a four-hour drive from the main hospital.
		(*e*) Describe any sensitivity analyses	NA	NA
**Results**				
Participants	13*	(a) Report numbers of individuals at each stage	NA	NA

		of study—eg numbers potentially eligible, examined for eligibility, confirmed eligible, included in the study, completing follow-up, and analysed		
		(b) Give reasons for non-participation at each stage	NA	NA
		(c) Consider use of a flow diagram	NA	NA
Descriptive data	14*	(a) Give characteristics of study participants (eg demographic, clinical, social) and information on exposures and potential confounders	10	Public hospitals in this survey are divided into two categories: primary healthcare facilities and secondary and tertiary healthcare facilities.<br>MPRs were only calculated if the medicine was available at a minimum of four facilities.<br>In view of the low availability of core medicines, we considered ten priority diseases to measure the affordability of standard treatments.
		(b) Indicate number of participants with missing data for each variable of interest	NA	NA
Outcome data	15*	*Cross-sectional study*—Report numbers of outcome events or summary measures	10	The availability of LPGs was 100% at the primary facilities. For IBs, however, only 11.5% were available in primary facilities.<br>The data also showed that the mean availability of LPGs and IBs was 32.6% and 36.8%, respectively, in secondary and tertiary facilities. Additionally, the mean availability of LPGs and IBs was 42.9% and 18.7%, respectively, in retail pharmacies.
Main results	16	(*a*) Give unadjusted estimates and, if applicable, confounder-adjusted estimates and their precision (eg, 95% confidence interval). Make clear which confounders were adjusted for and why they were included	NA	NA
		(*b*) Report category boundaries when continuous variables were categorized	10,11,12	In fact, only 7 LPGs in the secondary and tertiary facilities and 20 in the retail pharmacies had >50% availability. Additionally, only 24 IBs in the secondary and tertiary facilities and 4 in the retail pharmacies had >50% availability.<br>The MPRs of all LPGs ranged from 1.26 to 2.05, which demonstrated that the patient prices of LPGs approached the acceptable price line and were similar to the IRPs. However, the patient prices of IBs were all above the threshold level.<br>The cost of purchasing LPGs at all surveyed was between 0.1 and 0.8 days' wages,

				which indicated that generic medicines in Jiangsu Province were fairly affordable. Overall, IB products were less affordable than LPGs in both the public and private sectors.
		(*c*) If relevant, consider translating estimates of relative risk into absolute risk for a meaningful time period	NA	NA
Other analyses	17	Report other analyses done—eg analyses of subgroups and interactions, and sensitivity analyses	NA	NA
**Discussion**				
Key results	18	Summarise key results with reference to study objectives	12,13, 14,15，16	Our results indicate, first, the success of the essential medicine policy because the availability of LPGs in primary care facilities has reached 100%. According to statistics from the Ministry of Health in China, the visits to primary outlets has reached 3.92 billion by November 2014, accounting for 57.9% of the total visits. Therefore, the success has enabled the population who visit primary care facilities for common ailments to have reliable access to essential medicines after 2009. However, the availability of LPGs in secondary and tertiary facilities and private sectors remains relatively low. Little difference is found among medicine outlets when comparing the prices of LPGs to their IRPs.<br>The MPRs of medications on the core list of IBs available in both sectors were generally higher in the public sector than in the private sector.<br>The data from this survey show that most LPGs for standard treatments are affordable.<br>A comparative analysis of the affordability of IBs and LPGs indicates that the former are less affordable than the latter.
Limitations	19	Discuss limitations of the study, taking into account sources of potential bias or imprecision. Discuss both direction and magnitude of any potential bias	16	The present study has two limitations. First, the data are available only for the day they were collected at each facility in five cities of Jiangsu Province and may not reflect average monthly or yearly availability of medicines. Another is that calculating affordability based on unskilled government worker wages may lead to overly optimistic results because a portion of the national population earns less than that wage.
Interpretation	20	Give a cautious overall interpretation of results considering objectives, limitations, multiplicity of analyses, results from similar studies, and other relevant	12,13,14, 15	However, the availability of LPGs in secondary and tertiary facilities and private sectors remains relatively low. Minimal changes can be observed when compared the availabilities of LPGs in secondary and tertiary facilities for 21 common medicines

		evidence		surveyed in Shandong before the reform (28.82%) and in this survey after the reform (30.53%), which reflects a failure to implement the policy in these levels.<br>Little difference is found among medicine outlets when comparing the prices of LPGs to their IRPs.<br>This finding is similar to that in the studies conducted in Hubei and Shaanxi (core list MPRs were 1.05 and 1.84 for the public sector and 0.51 and 1.46 for the private sector in Hubei and Shaanxi, respectively).<br>In Thailand, analyses revealed that the MPR for IBs was higher in the private sector (11.60) than in the public sector (4.36). This difference in prices might be a reason for the difference in the healthcare policies of Thailand and China.
Generalisability	21	Discuss the generalisability (external validity) of the study results	15	Regarding the interpretation of affordability, caution should be exercised when extrapolating the findings to the national level because there may be regional differences in affordability due to differences economic development across the country.
**Other information**				
Funding	22	Give the source of funding and the role of the funders for the present study and, if applicable, for the original study on which the present article is based	17	This project is supported by personal financing rather than funding from official agencies.

Description: A completed checklist for the STROBE guidelines for observational studies, showing that this research adhered to the guidelines
